# Performance of a 74-Microhaplotype Assay in Kinship Analyses

**DOI:** 10.3390/genes15020224

**Published:** 2024-02-10

**Authors:** Carmen Tomas, Pedro Rodrigues, Carina G. Jønck, Zohal Barekzay, Halimureti Simayijiang, Vania Pereira, Claus Børsting

**Affiliations:** Section of Forensic Genetics, Department of Forensic Medicine, Faculty of Health and Medical Sciences, University of Copenhagen, Frederik V’s Vej 11, DK-2100 Copenhagen, Denmark; carmen.tomas@sund.ku.dk (C.T.); pedro.rodrigues@sund.ku.dk (P.R.); carina.joenck@sund.ku.dk (C.G.J.); zohalbarekzay@gmail.com (Z.B.); vania.pereira@sund.ku.dk (V.P.)

**Keywords:** microhaplotype, STR, SNP, forensic genetics, relationship testing, massively parallel sequencing, next generation sequencing

## Abstract

Microhaplotypes (MHs) consisting of multiple SNPs and indels on short stretches of DNA are new and interesting loci for forensic genetic investigations. In this study, we analysed 74 previously defined MHs in two of the populations that our laboratory provides with forensic genetic services, Danes and Greenlanders. In addition to the 229 SNPs that originally made up the 74 MHs, 66 SNPs and 3 indels were identified in the two populations, and 45 of these variants were included in new definitions of the MHs, whereas 24 SNPs were considered rare and of little value for case work. The average effective number of alleles (A_e_) was 3.2, 3.0, and 2.6 in Danes, West Greenlanders, and East Greenlanders, respectively. High levels of linkage disequilibrium were observed in East Greenlanders, which reflects the characteristics of this population that has a small size, and signs of admixture and substructure. Pairwise kinship simulations of full siblings, half-siblings, first cousins, and unrelated individuals were performed using allele frequencies from MHs, STRs and SNPs from Danish and Greenlandic populations. The MH panel outperformed the currently used STR and SNP marker sets and was able to differentiate siblings from unrelated individuals with a 0% false positive rate and a 1.1% false negative rate using an LR threshold of 10,000 in the Danish population. However, the panel was not able to differentiate half-siblings or first cousins from unrelated individuals. The results generated in this study will be used to implement MHs as investigative markers for relationship testing in our laboratory.

## 1. Introduction

Autosomal STRs have been the genetic markers of choice in forensic genetic investigations for more than two decades. STRs are highly polymorphic, which makes them valuable in both human identification and kinship investigations [1]. Other sets of genetic markers (e.g., Y-STRs, X-STRs, or SNPs) may be used for relationship testing and are often investigated in deficiency kinship cases. The introduction of massively parallel sequencing (MPS) techniques made it possible to genotype all the traditional forensic genetic loci in a single experiment [2]. MPS also facilitated new genotyping possibilities and a new set of loci, microhaplotypes (MHs), were suggested for forensic genetic case work. MHs were defined as short regions (200–300 bp) with two or more SNPs or indels that may be sequenced using standard MPS workflows [3]. The relative short distances between the variants allow for efficient PCR amplification and sequencing of the entire amplicon, which makes PCR-MPS assays targeting MH loci highly sensitive and potentially interesting for forensic genetic applications [4]. MHs have three important advantages compared to the standard STR loci: (1) Amplification of MHs does not generate stutter artefacts, which are made by polymerase slippage during the PCR amplification of tandem repeats [5] and complicates data analysis of mixture samples [1]. (2) The mutation rates of MHs are four to six orders of magnitude lower than the mutation rates of STRs [3], which is particularly important for relationship testing where mutations within a family may lead to interpretation ambiguity [6,7,8]. (3) The amplicon lengths of the different MH alleles are the same. This prevents an MPS read count variation due to different-sized alleles, which is observed for many STRs [9] and may be a problem in the analysis of highly degraded samples.

Several candidate MH loci [3,10,11,12,13,14,15,16,17] were identified via the 1000 genomes project [18]. They were tested on either Ion PGM [19], Ion S5 [11,20], NextSeq 500 [14,21], or MiSeq MPS platforms [11,12,13,16,22,23]. Two statistical parameters were typically used for the final selection of MH loci: the effective number of alleles, A_e_ [24], and the informativeness for assignment, I_n_ [25]. A high A_e_ indicates a relatively high variability, and that the MH locus is well suited for human identification, relationship testing, and analysis of mixtures. A high I_n_ indicates that the differences in MH allele frequencies between populations are relatively large and that the locus is useful for population assignment. The random match probabilities (RMPs) of recently published MH panels far exceed the RMPs of commonly used STR assays [11,12,13,14,15,20,22]. Furthermore, MH panels were effective assays for relationship testing [11,12,16,21,23] and mixture analysis [14,17,19,20].

We tested a customized PCR-MPS panel designed for the Ion S5 platform named the Ion AmpliSeq™ MH-74 Plex Research Panel, available in https://ampliseq.com (accessed on 8 January 2024) as a community panel [20]. The panel includes 38 and 30 MHs from the top 50 list of MHs with the highest A_e_ and I_n_ values, respectively, from a previous selection of MH loci [10,26]. The aims of this study were to: (i) test a set of 74 autosomal microhaplotypes in Danes and Greenlanders; to (ii) compare the information obtained from MHs to the marker sets used in our routine relationship case work, which include a set of 21 autosomal STRs and a set of 88 autosomal SNPs; to (iii) develop a strategy for analysis and reporting of MHs in relationship case work.

## 2. Materials and Methods

### 2.1. Samples

A total of 292 samples from presumably unrelated individuals were selected from the ”Section of Forensic Genetics anonymous collection of samples” (RAASP-D) (j. no. 004-0065/21-7000). Of these samples, 125 were from Denmark and 167 were from Greenland (79 of them were born in West Greenland and the other 88 in East Greenland). The samples were buccal cells collected on Whatman^®^ FTA^®^ cards (Merck KGaA, Darmstadt, Germany) or blood samples.

All samples were fully anonymized. The study follows the policy of the National Science Ethics Committee in Denmark (https://en.nationaltcenterforetik.dk; accessed on 8 January 2024) and complies with the rules of the General Data Protection Regulation (Regulation (EU) 2016/679).

### 2.2. DNA Extraction, DNA Quantification, and PCR Amplification

The DNA extraction was performed automatically using an EZ1^®^ Advanced XL robot (Qiagen, Hilden, Germany) together with an EZ1^®^ DNA investigator Kit (Qiagen) or manually, using a QIAamp DNA Investigator Kit (Qiagen). DNA concentration was measured using a Qubit^®^ 3.0 Fluorometer and a Qubit^®^ dsDNA HS Assay Kit (Thermo Fisher Scientific, Waltham, MA, USA).

The PCR amplification was performed using 10 µL of primer mix (Thermo Fisher Scientific), 4 µL of 5X Ion Ampliseq™ HiFi Mix (Thermo Fisher Scientific), and 0.2–1 ng of input DNA in 6 µL of molecular grade water. An Applied Biosystems^®^ Veriti^®^ 96-well thermal cycler (Thermo Fisher Scientific) was used with the following cycling program: 99 °C 2 min; (99 °C, 15 s; 60 °C, 4 min) × 24 times. The PCR primer mix was kindly provided by Thermo Fisher Scientific.

### 2.3. Library Building and DNA Sequencing

DNA libraries were prepared from amplicons using a Precision ID Library Kit (Thermo Fisher Scientific) and an Ion Express™ Barcode X kit (Thermo Fisher Scientific). Libraries were purified using Agencourt^®^ AMPure^®^ XP Reagents (Agencourt, Beverly, MA, USA), and quantified with the Qubit^®^ 3.0 Fluorometer using the Qubit^®^ dsDNA HS Assay Kit (Thermo Fisher Scientific). The purified libraries were diluted to a final concentration of 80 pM. For each sequencing chip, a pool with equimolar ratios of each sample was prepared. Emulsion PCR and loading of the chips were performed with an Ion Chef™ instrument (Thermo Fisher Scientific) and Precision ID Chef Reagents (Thermo Fisher Scientific). Sequencing was done on the Ion GeneStudio S5™ system (Thermo Fisher Scientific) using a Precision ID S5™ sequencing kit (Thermo Fisher Scientific), and Ion 530™ Chip Kits (Thermo Fisher Scientific). All reactions were performed following the manufacturer’s recommendations.

### 2.4. Haplotype Calling

Haplotypes were called using Torrent Suite v.5.10.1 software on an S5 Torrent Server VM (Thermo Fisher Scientific), together with a TVC_Microhaplotyper_v8.1 plugin (default settings) available by request from Thermo Fisher Scientific. The BED files for targets and hotspots were facilitated by Thermo Fisher Scientific and updated with 42 additional SNPs identified with the MHinNGS v1.0 software (see below). A supplementary quality check of the TVC_Microhaplotyper_v8.1 plugin results was performed by using an in-house developed Python script (mh_tvc). The mh_tvc script checks read depths and calculates the allele ratio (the ratio between the number of reads for the most frequent allele and the number of reads for the second most frequent allele). Genotypes were called as homozygotes, heterozygotes, or considered inconclusive for allele ratio values higher than 10, less than 3, or between 3 and 10, respectively. Homozygote genotypes were accepted when the number of reads was higher than 99. For heterozygote alleles, the minimum accepted number of reads was 50. Background noise was calculated as the ratio of the number of reads for the most frequent allele to the number of reads with different genotype calls from the genotype. Genotypes with a high background (background ratio < 10) were disregarded.

The FASTQ files were also analysed using a custom-made Python script named MHinNGS [27], which is freely available MH analysis software developed for the analysis of MHs in single-end sequencing data (https://hub.docker.com/r/bioinformatician/mhinngs; accessed on 8 January 2024). The configuration file with detailed analysis criteria for each MH locus is shown in Supplementary Table S1. Default analysis criteria [27] were used for most loci. However, the ”noise filter” was increased from 1% to a maximum of 2.5% for 7 loci, and the ”slide” function was increased from 2% to a maximum 5% for 32 of the 74 loci (Supplementary Table S1). These changes were introduced to overcome problems in homopolymer regions and simplify the manual data analysis. Haplotype calls were made independently by two analysts using the MHinNGS output files and the results were compared. If the haplotype calls differed, the analyses were repeated by both analysts. All samples were typed in duplicate and the results from the two experiments were compared. The haplotypes generated with MHinNGS were also compared to the haplotypes from the TVC_Microhaplotyper_v8.1 plugin.

An integrative genomics viewer (IGV v.2.7.2) tool [28] was used to visualize and confirm some of the haplotypes.

### 2.5. Population Genetic Analyses

Arlequin ver 3.5.2 [29] was used to perform calculations on population statistical parameters including allele frequencies, observed (H_o_) and expected (H_e_) heterozygosity, the Hardy–Weinberg equilibrium (HWE), the linkage disequilibrium (LD), and pairwise *F*_ST_ values. The *p*-values were assessed in Arlequin using 1 million steps in the Markov chain and 1 million dememorization steps, for HWE and LD tests, and 10,000 permutations, for the *F*_ST_ calculations. The Holm–Šidák method was used for the correction of multiple statistical tests [30].

The effective number of alleles (A_e_) was calculated as A_e_ = 1/(1 − H_e_) as described by Crow and Kimura (1970) [24].

### 2.6. Simulations and LR Calculations

Simulated profiles were generated and analysed using the ”simulate” and ”likelihood” options of the Merlin 1.1.2 software [31] together with an in-house developed Python script (merlin_converter_lr). A total of 1000 pedigree files were generated for each of the following scenarios: two full siblings, two half-siblings, two cousins, and two unrelated individuals. The likelihood ratio LR = P(MHs|H_1_)/P(MHs|H_2_) was calculated for all simulated pairs, where H_1_ = the pair of individuals that were related (siblings, half-siblings, or cousins) and H_2_ = the pair of individuals that were unrelated. The Log_10_(LR) was represented and the typical LR was calculated for each set of simulations. Allele frequencies for 21 STRs based on 1335 Danes ([32]; unpublished data), 21 STRs based on 519 Greenlanders [32,33], 88 SNPs based on 82 Danes [34], 39 SNPs based on 164 Greenlanders ([35]; unpublished data), and 72 MHs based on 125 Danes or 167 Greenlanders (this study) were used to generate the profiles. The genetic distance between genetic markers was obtained from a Rutgers map v.3 [36].

## 3. Results

### 3.1. Sequencing Results and Haplotype Calling

A total of 125 samples from Danish individuals and 167 samples from Greenlanders were genotyped in duplicate using a customized 74-plex microhaplotype assay on the Ion S5 platform. The samples were sequenced in 12 runs with 32–38 samples per Ion 530 chip. The total number of reads per sample averaged 233,828 reads for the 292 genotyped samples.

The FASTQ files were analyzed with MHinNGS software that was developed for the analysis of well-defined MHs [27]. In addition to the 229 SNPs that were originally described in the 74 MH loci [20], 66 SNPs and 3 indels were identified in the Danish and Greenlandic individuals (Supplementary Table S2). Of these variants, 42 SNPs and 3 indels were included in new definitions of the MHs and in the MHinNGS configuration file (Supplementary Table S2). Thus, MHinNGS would identify 271 SNPs and 3 indels in 74 MH loci and name the MH alleles according to these variants. A total of 24 SNPs were only observed once or twice in the two populations and were defined as ”rare SNPs” in the MHinNGS configuration file (Supplementary Table S1). ”Rare SNPs” are not part of the MH *per se* and are not included in the MH allele name. However, when the rare variant is observed, a flag will appear in the comment column of the MHinNGS result file [27]. Furthermore, nine linked variants were identified (Supplementary Table S2). These variants appeared to be linked to specific MH alleles and did not add any novel identifying information. Nevertheless, linked variants were defined in the MHinNGS configuration file (Supplementary Table S1) to simplify the manual data analysis [27].

On some fragments, there were specific positions where a relatively high fraction of the reads has another base call than that of most of the reads; this may be observed in many individuals [37]. The ambiguous base calls generate multiple unique sequences that can be eliminated by replacing the base call with an ”N” using the ”ignore position” criterion in MHinNGS [27]. Of the 17,653 nucleotides sequenced with the 74-plex MH panel, 113 positions (0.6%) were ignored, and another 140 possible single nucleotide insertions were ignored (Supplementary Table S1). The latter were introduced to overcome errors around homopolymer regions. Ignoring these positions increased the read depth of the alleles, simplified analysis, and prevented reporting of questionable base calls.

The sequencing data were also analyzed with the Torrent Suite v.5.10.1 and the TVC_Microhaplotyper_v8.1 plugin that performs variant calling by identifying the genotypes at the positions specified in the BED files. The plugin could not genotype indels or report new variants. However, we updated the hotspot BED file with the 42 SNPs identified with MHinNGS and, thus, the TVC_Microhaplotyper_v8.1 plugin called the genotypes of 271 SNPs in the 74 MHs. Acceptance criteria were imposed on the genotyping results from the plugin using the in-house developed Python script mh_tvc (see Section 2) and complete concordance with the genotypes called by MHinNGS was obtained.

Two loci, mh03KK-150 and mh09KK-033, were removed from all downstream analyses. The mh03KK-150 locus showed allele dropouts and inconsistent results were observed between duplicate runs, while mh09KK-033 suffered from locus dropout in more than 70% of the samples, mainly from Greenland. Of the 21,024 possible MH genotypes, 21,013 (99.95%) were called, and only 11 MH genotypes (0.05%), all in Greenlandic samples, did not meet the criteria established for MH genotype calling.

### 3.2. Population Genetics Parameters

The MH allele frequencies for Danes and Greenlanders are shown in Supplementary Table S3. In the Danish population, 387 MH alleles were identified, while fewer MH alleles were found in the populations from West and East Greenland, 366 and 337 respectively. The average A_e_ for all loci (Supplementary Table S4) was also higher in Danes (A_e_ = 3.2) than in West Greenlanders (A_e_ = 3.0) and East Greenlanders (A_e_ = 2.6). Only four loci (mh02KK-134, mh11KK-180, mh18KK-213, mh18KK-218) displayed A_e_ values higher than 6 in the Danish population, and just one (mh13KK-213) in West Greenlanders. In the East Greenlandic population, no loci had A_e_ values higher than 6. Several loci had A_e_ values ranging from 3 to 6. Danes presented 30 loci, West Greenlanders 28 loci, and East Greenlanders 20 loci with A_e_ values in that range. Overall, mh18KK-218, mh02KK-134, mh18KK-213, and mh11KK-180 were the most polymorphic loci in the three studied populations (Supplementary Table S4). The loci with low A_e_ values varied between the tested populations. In Danes, one monomorphic locus (mh15KK-095) was found and three other loci (mh05KK-123, mh05KK-122, and mh16KK-053) had A_e_ values next to 1. Both Greenlandic populations shared the same three loci (mh17KK-105, mh12KK-202, mh02KK-201) with the lowest A_e_ values. Interestingly, some of the loci with low A_e_ seemed promising for a future ancestry MH kit, especially mh05KK-123 and mh05KK-122, that presented very distinct allele frequencies in the studied populations.

No statistically significant *(p* < 0.05) deviations from Hardy–Weinberg equilibrium were observed after Holm–Šídák correction for any of the tested markers or populations.

LD was assessed for all the pairs of microhaplotypes in each population. After Holm–Šídák correction, two pairs of microhaplotypes showed statistically significant (*p* < 0.05) LD in the Danish dataset. In West and East Greenlanders, statistically significant (*p* < 0.05) LD values were observed in 5 and 54 pairs of loci, respectively. Higher levels of LD among Greenlanders, and especially in East Greenland, have previously been described [33,38]. LD can be attributed to several population factors, such as inbreeding, population admixture or population structure. The small population size and the historical events in the Greenlandic population could explain the high levels of LD, that have also been reported for other genetic markers [39].

Pairwise *F*_ST_ values were calculated between the three studied populations (Supplementary Table S5). High and statistically significant (*p* < 0.05) distances were observed for all population pairs. For the population in Greenland, 2.3% of the variability was due to differences between West and East Greenlanders. Significant differences had previously been observed for these two groups analysed with other markers sets [33,40,41].

### 3.3. Kinship Simulations and LR Calculations

Pairwise kinship simulations were performed using allele frequencies from MHs, STRs and SNPs from Danish and Greenlandic populations as described in Section 2.6. For this analysis, the MH allele frequency data from West and East Greenland were merged to make them comparable to the STR and SNP frequencies from Greenlanders that were previously obtained in a single Greenlandic population [32,33,35]. Only frequencies for 39 SNPs were available for the Greenlandic population, whereas 88 SNPs were available for Danes, and only 53 MHs were used for the simulations of Greenlandic relationships to avoid pairs of markers in LD.

**Figure 1:**
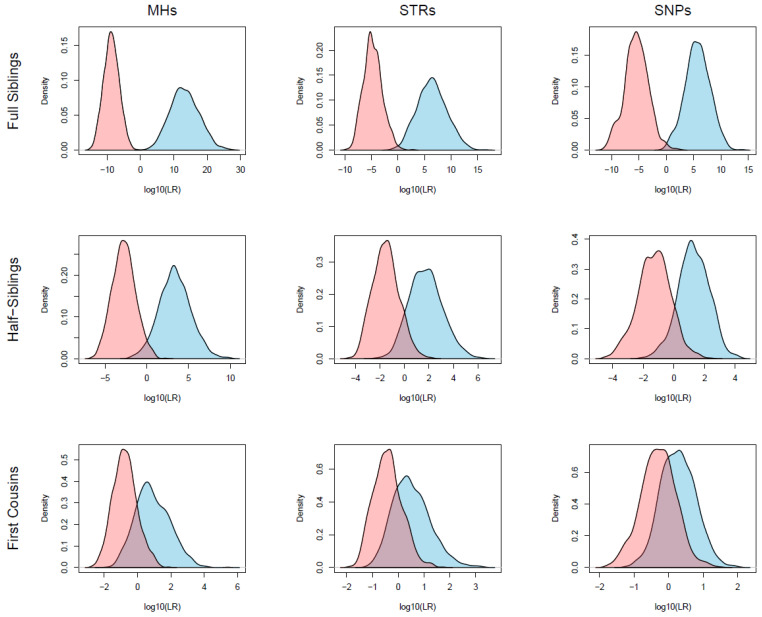
Distributions of Log_10_(LR) calculated using 72 MHs, 21 STRs and 88 SNPs for full siblings, half-siblings, and first cousins in Danes. For each kinship scenario, 1000 simulations of pairwise relationships were obtained. Log_10_(LR) were drawn for true relationships (blue curve) and true unrelated pairs (pink curves).

Simulations and LR calculations were done for three different degrees of kinship scenarios: full siblings (first-degree), half-siblings (second-degree), and first cousins (fourth-degree), and for unrelated individuals. Figure 1 and Supplementary Figure S1 show the Log_10_(LR) distributions of these relationships in the Danes and Greenlanders, respectively, based on the three sets of markers. Table 1 shows the typical LRs obtained from the simulations, and Table 2 (Danes) and Supplementary Table S6 (Greenlanders) show the false positive and false negative rates using different LR thresholds.

In both Danes and Greenlanders, typical LR values for simulated relationships using MHs far exceeded the typical LR values obtained with STRs and SNPs. As expected, typical LRs were highest for siblings and lowest for half-siblings and cousins.

For simulated siblings, the LRs based on MHs ranged from 12.9 to 6.31 × 10^26^ in Danes and from 0.8 to 4.33 × 10^19^ in Greenlanders. The LR distributions for simulated siblings and unrelated pairs did not overlap in Danes (Figure 1), which indicates that the 72 MHs may differentiate siblings from unrelated individuals in most cases. For the STR and SNP panels, there were some overlaps in the LR distributions in Danes, and the false positive rates were around 1% with a LR threshold of one (Table 2). In Greenlanders, there was a small overlap in LR distributions for simulated siblings and unrelated pairs. However, these simulations were made with fewer MH loci. Similarly, fewer SNPs were used, and the false positive rate for the SNP panel was more than 5% using an LR threshold of one (Supplementary Table S6).

In the analysis of simulated half-sibling pairs, the LR values obtained from the MHs ranged from 0.01 to 6.47 × 10^9^ in the Danish population and from 0.02 to 2.26 × 10^8^ in Greenlanders. There was a clear overlap between the LR distributions from simulated half-siblings and unrelated pairs (Figure 1 and Supplementary Figure S1) and the false positive rate was 3.2% and 5.7% in Danes and Greenlanders, respectively, with an LR threshold of one (Table 2 and Supplementary Table S6). For the STR and SNP panels, the overlap between LR distributions and the false positive rates were even larger. For all marker sets, it required an LR of at least 1000 to reduce the false positive rate to 0%. However, if the LR threshold was set to 1000, the false negative rates were 42%, 81%, and 96% of the true half-siblings using MHs, STRs, and SNPs, respectively, in Danes, and 74%, 90%, and 100% in Greenlanders.

When analysing simulated cousin pairs, the overlaps between LR distributions were considerable and the false positive rates were higher than 15% for all marker sets with an LR threshold of one (Table 2 and Supplementary Table S6). The false negative rates were also very high and LRs were rarely higher than 100 for true simulated cousin pairs with any of the markers sets.

## 4. Discussion

This study demonstrates that MHs are valuable markers for relationship testing. The 72 MH assay was able to resolve all simulated sibling cases in Danes, something that was not possible with the STR and SNP panels currently used in our laboratory, even when both sets of markers were combined. Even the 53 MHs used for the simulation of Greenlandic siblings provided more information and higher evidential weights than the STR and SNP panels. For the more distant relationships of simulated half-siblings and cousins, it was clear that neither of the panels were able to resolve these cases, and that more markers will be needed.

It was previously estimated that around 200 MHs with A_e_ > 4.5 would be necessary to resolve second-degree relationships (e.g., half-siblings), whereas third- (e.g., uncle/aunt and nephew/niece) and fourth-degree relationships (e.g., first cousins) would require many hundreds or even thousands of MHs [16,21]. For these types of relationships, other panels with a much higher number of markers, e.g., the FORCE panel with more than 5400 SNPs [42], may be applied. In this work, we used STR allele frequencies from PCR-CE analyses for the simulations and it may be speculated that sequenced STR alleles may provide more information. However, data from STR sequencing would only have marginal effects on the evidential weights [43].

In our laboratory, all samples in relationship cases are typed for 21 autosomal STRs ([32,33]; unpublished data). Supplementary investigations may involve genotyping of 88 autosomal SNPs [34], 16 Y-STRs [40,44], 12 X-STRs [38], or whole genome mtDNA [45], depending on the relationship query in question. Our laboratory investigates approximately 600 relationship cases every year and between 5 and 7% of these usually require supplementary investigations. Based on the results obtained in this work, we plan to validate and implement the MH assay as a supplementary investigation in the near future. The MH assay will likely replace the Precision ID Identity panel [34] as the primary supplementary assay.

The MH allele frequencies for Danes may be used for evidential weight calculations in cases involving Danish and European individuals. For Greenlanders, the results indicate that the MH allele frequencies differ significantly between West and East Greenland, which corroborates previous studies of autosomal STRs [33], Y chromosome [40], and mtDNA markers [41]. Moreover, statistically significant (*p* < 0.05) *F*_ST_ distances were observed between the two Greenlandic regions. Therefore, two allele frequency databases are required for kinship investigations of Greenlanders. Alternatively, a single Greenlandic allele frequency database can be used if the calculations are adjusted using theta correction. High levels of LD were observed between MH pairs in the Greenlandic population, especially in the Eastern region. When the number of genetic markers increases, the probability of finding pairs of markers in LD increases as well, especially in small and sub-structured populations. Inheritance of markers in LD does not occur independently and the multiplication rule cannot be applied. As far as we know, none of the available kinship software developed for the analyses of linked markers takes LD into account, due to the complexity of the calculations. This is, for example, the case of FamLink [46], the software that our laboratory plans to implement for relationship cases investigated with MHs. We will therefore only use one of the MHs that are in LD (the one with the highest A_e_ value) for the calculation of the evidential weight. The markers not included in the calculations will only be considered if they show genetic inconsistencies in the investigated relationship.

The conditions used for genotyping in this work (0.2–1.0 ng DNA input, 24 PCR cycles, 80 pM library pool, 32–38 samples per Ion 530™ Chip) generated almost complete profiles for all samples. We conducted a small sensitivity study, which indicated that lower DNA inputs led to low read depths and a failure to fulfil the criteria defined for MHinNGS and the mh_tvc python script in some loci, although the genotypes were accepted using the TVC_Microhaplotyper_v8.1 plugin. The criteria for genotyping come from previous experiences with SNP typing [34] and generated robust genotype calls for case work and in proficiency tests. Thus, a lower limit of 200 pg DNA input is recommended for the MH assay, which is similar to the sensitivity of previously evaluated PCR-MPS assays in our laboratory [9].

MHs are promising new markers for forensic genetics and may have applications in relationship testing, human identification, mixture deconvolution, and population assignment. The panel of 74 MHs used here was selected from early studies of MHs [10,26] and has a dual purpose of human identification and population assignment. Thus, not all the MHs in the panel are ideal for relationship testing *per se*. More informative markers with higher A_e_ have since then been identified [11,12,13,14,15,16,17] and registered in the MicroHapDB database [47]. After the congress of the International Society of Forensic Genetics (ISFG) in 2022, a *MH working group* was formed with participants from forensic genetic laboratories and interested companies. The purpose of the working group is to define the parameters for selecting a core panel of MHs and to provide the framework for a future ISFG commission on MHs that may recommend a formal set of MH markers for forensic genetic testing. Until this work is completed, the 74 MH assay provides a useful investigation for relationship queries and human identification.

## Figures and Tables

**Table 1:** Typical LRs for pairs of full siblings, half-siblings, and first cousins.

Marker Sets	Denmark			Greenland		
	Full Siblings	Half-Siblings	First Cousins	Full Siblings	Half-Siblings	First Cousins
MHs *	1.87 × 10^13^	2412	8	4.62 × 10^8^	147	4
STRs **	2.55 × 10^6^	57	3	2.04 × 10^5^	30	3
SNPs ***	5.54 × 10^5^	19	2	168	3	1

* Based on 72 MHs in Danes and 53 MHs in Greenlanders. ** Based on 21 STRs. *** Based on 88 SNPs in Danes and 39 SNPs in Greenlanders.

**Table 2:** False positives and negatives rates obtained for simulated data in Danes.

Relationship	LR Threshold	MH		STR		SNP	
		False Positives †	False Negatives ‡	False Positives †	False Negatives ‡	False Positives †	False Negatives ‡
**Siblings**	**1**	0.00%	0.00%	0.70%	0.80%	1.00%	0.20%
	**10**	0.00%	0.00%	0.20%	1.70%	0.40%	2.20%
	**100**	0.00%	0.10%	0.10%	5.30%	0.10%	5.60%
	**1000**	0.00%	0.30%	0.00%	11.80%	0.00%	11.00%
	**10,000**	0.00%	1.10%	0.00%	18.80%	0.00%	21.10%
**Half-siblings**	**1**	3.10%	3.60%	8.80%	9.40%	11.40%	10.00%
	**10**	0.30%	10.00%	1.30%	30.90%	2.00%	38.30%
	**100**	0.10%	24.00%	0.20%	57.00%	0.20%	75.20%
	**1000**	0.00%	41.60%	0.00%	81.40%	0.00%	96.30%
	**10,000**	0.00%	63.90%	0.00%	94.10%	0.00%	99.60%
**Cousins**	**1**	14.40%	18.30%	22.70%	26.00%	27.70%	31.00%
	**10**	1.00%	56.60%	1.40%	76.10%	1.10%	92.20%
	**100**	0.00%	84.50%	0.00%	96.60%	0.00%	99.90%
	**1000**	0.00%	96.90%	0.00%	99.70%	0.00%	100.00%
	**10,000**	0.00%	99.70%	0.00%	100.00%	0.00%	100.00%

† False positives: number of LR values higher than the LR limit when unrelated = true/number of unrelated (1000). ‡ False negatives: number of LR values below the LR limit when related = true/number of related (1000).

## Data Availability

The data presented in this study are available in article and supplementary material.

## Supplementary Materials

The following supporting information can be downloaded at: https://www.mdpi.com/article/10.3390/genes15020224/s1, Supplementary Figure S1: Distributions of Log_10_(LR) calculated using 53 MHs, 21 STRs and 39 SNPs for full siblings, half-siblings, and first cousins in Greenlanders. For each kinship scenario, 1000 simulations of pairwise relationships were obtained. Log_10_(LR) were drawn for true relationships (blue curve) and true unrelated pairs (pink curves); Supplementary Table S1: Configuration file for MHinNGS analysis; Supplementary Table S2: SNPs present in the 74-plex with new variants, rare SNPs, and linked alleles; Supplementary Table S3: Allele frequencies for 72 MHs in Denmark, East Greenland, and West Greenland; Supplementary Table S4: Population data (observed and expected heterozygosity, allele count, and A_e_) for Danes, West Greenlanders, East Greenlanders; Supplementary Table S5: Pairwise *F*_ST_ values calculated for all population pairs based on 72 MHs; Supplementary Table S6: False positive and negative rates obtained for simulated data in Greenlanders.
