# Supplementary figures and images for: Performance of a 74-Microhaplotype Assay in Kinship Analyses
Source: Genes (Basel). 2024 Feb 10;15(2):224. doi: 10.3390/genes15020224 (PMC10888013; doi:10.3390/genes15020224)

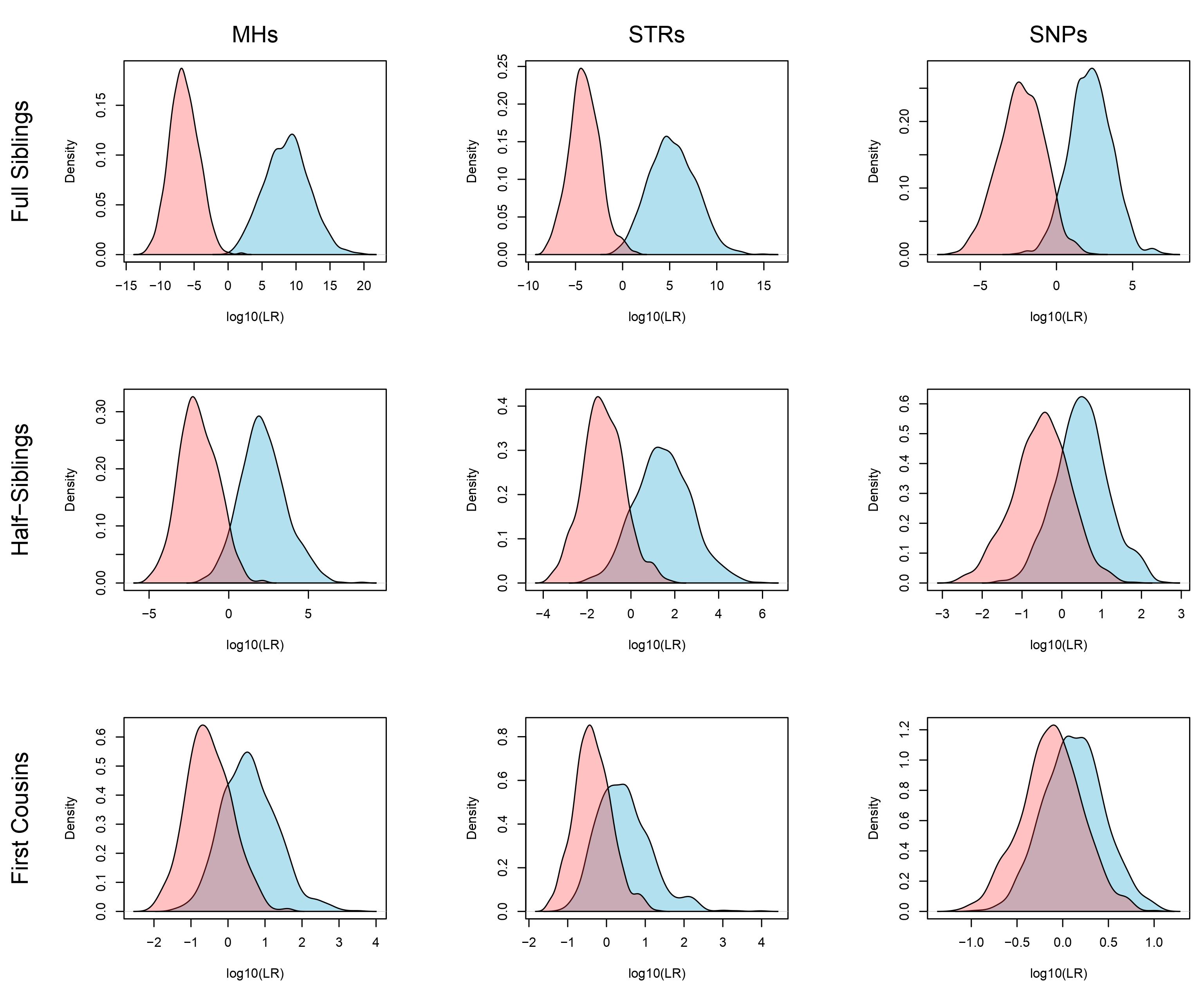

Supplement: Supplementary file 1 [file genes-15-00224-s001.zip › Supplementary Figure S1- GRL_curves.jpg]
